# A Regional Audit on Venous Thromboembolism (VTE) Prophylaxis Compliance and Cultural Considerations in Prescribing Porcine-Derived Low-Molecular-Weight Heparins

**DOI:** 10.7759/cureus.100617

**Published:** 2026-01-02

**Authors:** Minahil Mujahid, Husain Ahmed, Shibbir Ahmad, Isra Mujahid, Junaid Ur-Rehman, Geeta Maheshwari, Mohsen Norouzi, Ben Prudon

**Affiliations:** 1 Ophthalmology, Newcastle University Hospitals NHS Foundation Trust, Newcastle Upon Tyne, GBR; 2 Trauma and Orthopaedics, Newcastle University Hospitals NHS Foundation Trust, Newcastle Upon Tyne, GBR; 3 Anaesthesia, Royal Victoria Infirmary, Newcastle Upon Tyne, GBR; 4 Vascular Surgery, Royal Blackburn Teaching Hospital, Blackburn, GBR; 5 ENT, Darlington Memorial Hospital, Darlington, GBR; 6 Acute Medicine, University Hospital of North Tees, Stockton-on-Tees, GBR; 7 Haematology, South Tees Hospitals NHS Foundation Trust, Middlesbrough, GBR; 8 Respiratory Medicine, University Hospital of North Tees, Stockton-on-Tees, GBR

**Keywords:** autonomy, dietary compliance, doctor – patient, halal, lmwh, nice ng89, patient counselling, porcine-origin, vegan, vte compliance

## Abstract

Background: Hospital-acquired venous thromboembolism (VTE) remains a leading cause of preventable mortality in the NHS. NICE guidelines (NG89) mandate timely risk assessment and prophylaxis, yet practice varies widely. Many low-molecular-weight heparins (LMWHs), such as tinzaparin, are porcine-derived, posing ethical challenges for patients with specific dietary or religious beliefs.

Objective: The objective of this study is to evaluate adherence to NICE NG89 guidelines on VTE prophylaxis across two hospitals, with a particular focus on culturally sensitive prescribing. This audit also assessed clinician awareness of LMWH origins and explored patient preferences around dietary compliance. The impact of targeted interventions was measured across two audit cycles.

Method: A prospective two-cycle audit was conducted at two NHS hospitals in North East England, The James Cook University Hospital (JCUH) and University Hospital North Tees (UHNT), between March 2024 and January 2025. Patients with dietary requirements were identified via catering records and surveyed. A doctor questionnaire assessed awareness and counselling practice. Interventions included staff education, posters, prescribing alerts, and community outreach.

Results: Cycle one included 76 patients. Cycle two included 61 patients. VTE risk assessment improved by 14% at UHNT; JCUH maintained 100% compliance. However, timely prophylaxis fell in UHNT, with 80% receiving it late. Documentation and discussions regarding VTE and dietary compliance improved but remained suboptimal. Leaflet distribution rose by 10% at JCUH but was absent at UHNT. Notably, 98% (n = 137) of patients prioritised medication aligned with their beliefs, and nearly half would decline porcine-derived prophylaxis even if it prevented serious illness. Clinician questionnaire data showed 64% (n = 50) were unaware that LMWHs are animal-derived, and 94% (n = 50) did not inform patients, citing time pressures and limited knowledge.

Conclusion: While guideline compliance improved following intervention, particularly for risk assessments and documentation, major gaps remain in patient communication and dietary-informed prescribing. Most patients expected transparency and alternatives to porcine-based medications. Future efforts must embed dietary screening into routine prescribing and strengthen shared decision-making through structured education and culturally competent communication strategies.

## Introduction

Venous thromboembolism (VTE), comprising deep vein thrombosis and pulmonary embolism, is a preventable cause of morbidity and mortality in hospitalised patients. In the UK, VTE accounts for approximately 25,000 deaths annually [1]. NICE guidance NG89 and Quality Standard QS201 [2,3] set out standards for timely risk assessment and prophylactic intervention, with a national benchmark of 95% compliance within 14 hours of admission.

Pharmacological prophylaxis typically involves low-molecular-weight heparins (LMWHs), such as tinzaparin, which is porcine-derived [4]. This has important implications for patients who may be opposed to using pork products or consuming animals on religious, cultural, or ethical grounds. In the UK, significant proportions of the population identify with faiths such as Islam and Judaism, which prohibit the consumption of pork, while others may follow vegetarian or vegan diets [5,6]. However, there is a synthetic alternative, fondaparinux, which has comparable efficacy to LMWHs in similar clinical scenarios [7]. However, awareness and practice around this recommendation are inconsistent [8], and it may be more costly if used widely [9].

Respect for patient autonomy and informed consent is a core ethical principle in medical practice. The General Medical Council’s Good Medical Practice guidance states that clinicians must work in partnership with patients and support them in making informed decisions about their care [10]. This includes providing relevant information about proposed treatments, particularly where treatment components may conflict with patients’ beliefs or values. Failure to discuss the origin of medications such as LMWHs may therefore undermine informed consent and shared decision-making.

Existing literature examining physicians’ awareness of the porcine origin of LMWHs and their communication with patients regarding this issue is limited. A study conducted in North Bristol NHS Trust identified a significant gap in clinicians’ awareness of the animal-derived content of LMWHs and its ethical implications for patients with religious or dietary restrictions [11]. The study revealed that many healthcare professionals were unaware of the porcine origins and few routinely engaged in discussions with patients about this. The project showed that incorporating patients’ beliefs and values into prescribing decisions, especially concerning the composition of medications, led to enhanced patient engagement, improved trust in clinical care, and a more consistent approach to culturally competent prescribing. Despite these insights, a PubMed literature search reveals fewer than 10 studies directly addressing this issue, underscoring the imperative need for further research and structured educational interventions to support ethical, inclusive prescribing practices.

The James Cook University Hospital (JCUH) and University Hospital of North Tees (UHNT) undertake the first regional study in the North East focusing on the dietary requirements of patients who receive VTE prophylaxis. The primary objective of this audit was to evaluate compliance with national VTE guidelines in both hospitals and understand how important it is for patients for their medications to be compliant with their dietary preferences and determine clinicians’ awareness of the animal origins of LMWHs and their practices regarding patient consent and discussion of alternatives.

## Materials and methods

Study design

This was a prospective, two-cycle quality improvement audit conducted in accordance with NICE Guidance NG89 and Quality Standard QS201 [2,3]. The same methodology was applied in both audit cycles, with an intervention phase implemented between cycles. The project was registered with the audit departments at both participating sites.

Cycle one data were collected between March and June 2024. Following the implementation of targeted interventions, cycle two data were collected between October 2024 and January 2025.

Setting

The study was conducted at two NHS hospitals in the North East of England: James Cook University Hospital (JCUH) and University Hospital of North Tees (UHNT). Adult medical and surgical inpatient wards were included. Paediatric wards and intensive care units were excluded.

A multidisciplinary project team led the audit, comprising consultant clinicians, resident doctors, audit department staff, and representatives from the hospital catering teams, who supported the identification of patients with documented dietary requirements.

Participants

Patient Sample

Eligible participants were adult inpatients admitted to medical or surgical wards at JCUH or UHNT who had documented dietary requirements. Dietary needs were identified using ward-based catering records, which documented requirements such as halal, vegetarian, vegan, and specific medical diets (e.g., low-potassium). Eligibility was confirmed directly with patients prior to inclusion.

Across both cycles, 137 patients were included. Cycle one comprised 76 patients (51 at JCUH and 25 at UHNT), and cycle two comprised 61 patients (51 at JCUH and 10 at UHNT).

Doctor Sample

A separate cohort of 50 doctors was surveyed to assess clinician awareness of the porcine origin of LMWHs and counselling practices related to this issue. The questionnaire was distributed online to doctors ranging from foundation year 1 to consultant level.

Patient Questionnaire

Eligible patients were approached on the wards and invited to participate following verbal consent. A structured questionnaire was administered to collect data on VTE risk assessment, pharmacological prophylaxis prescribing and timing, documentation practices, patient recall of discussions regarding VTE prophylaxis, provision of patient information leaflets, and attitudes towards dietary compatibility of prescribed medications.

Following completion of the questionnaire, patients’ medication charts were reviewed to determine whether prescribed VTE prophylaxis aligned with their stated dietary preferences. All patient data were anonymised prior to analysis.

Doctor Questionnaire

An online questionnaire was developed to assess clinician awareness of the porcine origin of LMWHs, familiarity with alternative agents such as fondaparinux, and routine practices regarding patient discussion and consent. The questionnaire was disseminated to 50 doctors over a two-week period between June and July 2024. Responses were collected anonymously.

Interventions

Between audit cycles, a series of interventions were implemented across both hospital sites.

Educational sessions were delivered to a wide range of healthcare providers, including resident doctors, consultants, nurses, and pharmacists. These sessions took place during grand rounds, structured teaching programmes for foundation year doctors, and departmental teaching in medical and surgical wards.

**Figure 1:**
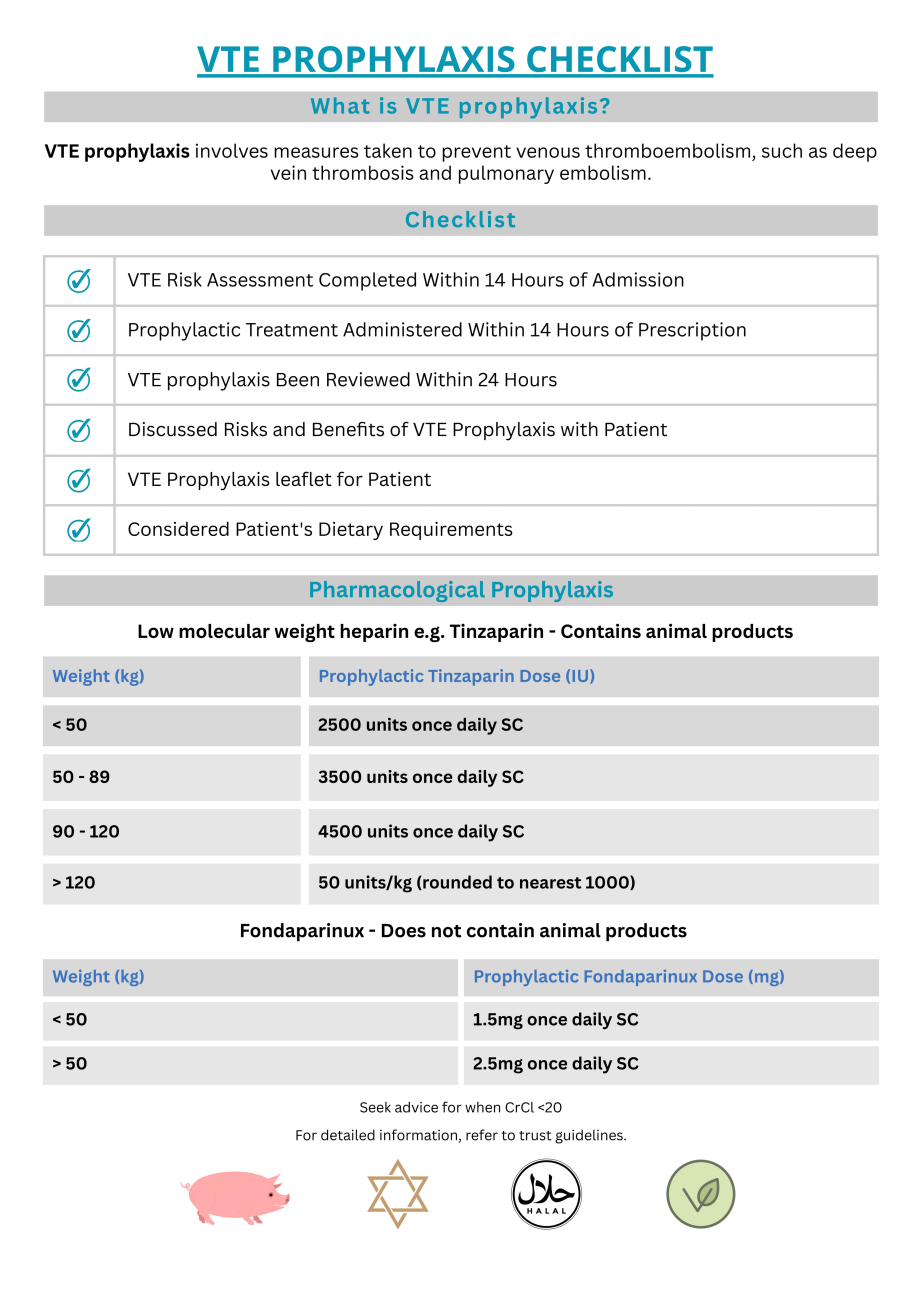
Leaflet Distributed to Raise Awareness of VTE Prophylaxis Assessment and Porcine Origin of LMWHs VTE: Venous thromboembolism; LMWHs: low-molecular-weight heparins Created by the authors

Informative posters (Figure 1) were designed and displayed across both sites, including acute wards, surgical units, and doctors’ staff rooms. The posters served as visual reminders to complete VTE risk assessments and provided guidance on LMWH dosing by weight category [12]. They also highlighted the porcine origin of LMWHs and outlined the availability and dosing of the non-porcine alternative.

Patient information leaflets on VTE prophylaxis were sourced from the local NHS foundation trust [13], printed, and distributed to wards at JCUH. These aimed to enhance patient understanding and support informed decision-making. However, the leaflets were only available in English.

Mandatory E-learning content was updated in collaboration with module developers to include specific reference to the porcine origin of LMWHs. These updates were incorporated into the VTE training module completed by all incoming trainees.

Electronic prescribing alerts were reviewed with local IT teams. JCUH had an existing digital reminder system prompting clinicians to complete VTE risk assessments on admission and at 14 hours. At UHNT, discussions were held regarding reinstatement of a similar system; however, concerns regarding disruption to emergency prescribing workflows led to a decision not to reintroduce alerts at that site.

Community engagement was undertaken with local faith groups, including imams and gurus. These discussions centred on increasing patient awareness of the porcine origin of common hospital medications and encouraging patients to advocate for treatments aligned with their values. This initiative aimed to empower patients to make informed, culturally respectful healthcare decisions [14].

Data analysis

All questionnaire and audit data were anonymised and entered into Microsoft Excel for descriptive analysis. Results were compared between audit cycles to assess changes following implementation of interventions.

## Results

Patient cohort

Cycle one comprised 76 patients (James Cook University Hospital (JCUH), n = 51; University Hospital of North Tees (UHNT), n = 25), while cycle two comprised 61 patients (JCUH, n = 51; UHNT, n = 10), yielding a total cohort of 137 patients across both audit cycles. The mean age of the combined cohort was 58 years.

Across both cycles, the predominant religious affiliation was Islam (cycle one: 75%, n = 57; cycle two: 59%, n = 36). Other recorded affiliations included Hinduism (cycle one: 12%, n = 9; cycle two: 8%, n = 5), as well as a composite “other” category encompassing Christian, Sikh, Buddhist, atheist, or no stated religious affiliation (cycle one: 13%, n = 9; cycle two: 33%, n = 13).

Documented dietary requirements reflected the observed religious distribution. Halal dietary requirements were most frequently recorded in both cycles (cycle one: 61%, n = 46; cycle two: 61%, n = 37). Vegetarian diets accounted for 26% (n = 20) of patients in cycle one and 30% (n = 18) in cycle two. Other dietary requirements, including vegan diets and avoidance of specific animal products such as pork or beef, were documented in 13% (n = 10) of patients in cycle one and 8% (n = 6) in cycle two.

VTE prophylaxis prescribing patterns

In cycle one, tinzaparin was the most frequently prescribed pharmacological agent for VTE prophylaxis, administered to 62% of patients (n = 47). Alternative prophylactic strategies included direct oral anticoagulants or alternative LMWH preparations (14%, n = 11), absence of pharmacological prophylaxis (12%, n = 9), thromboembolic deterrent stockings alone (7%, n = 5), and fondaparinux (5%, n = 4).

**Figure 2:**
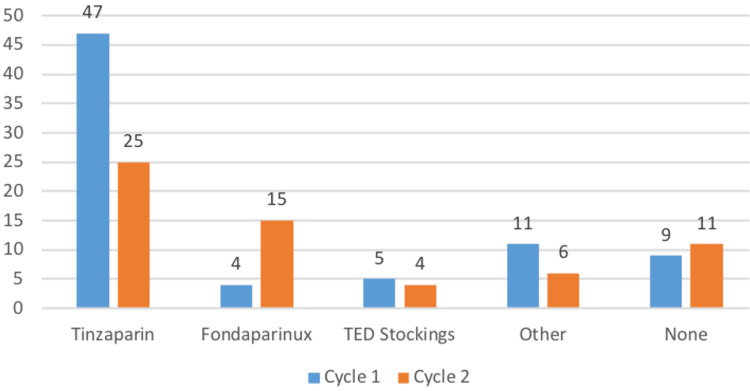
Distribution of Venous Thromboembolism Prophylaxis Modalities Across Two Audit Cycles

In cycle two, tinzaparin remained the most commonly prescribed pharmacological agent; however, its utilisation decreased to 41% of patients (n = 25). Conversely, the proportion of patients prescribed fondaparinux increased to 25% (n = 15), representing an absolute increase of 20 percentage points compared with cycle one. In this cycle, fondaparinux emerged as the second most frequently prescribed pharmacological VTE prophylaxis among patients with documented dietary requirements (Figure 2).

Compliance with NICE VTE prophylaxis standards

Compliance with key NICE NG89 standards is summarised in Table 1.

**Table 1:** Compliance with VTE Prophylaxis Guidelines (Assessment and Administration) by Hospital and Audit Cycle VTE: Venous thromboembolism; JCUH: James Cook University Hospital; UHNT: University Hospital of North Tees

	JCUH cycle one	JCUH cycle two	UHNT cycle one	UHNT cycle two
VTE risk assessment within 14 hours	100% (n = 51)	100% (n = 51)	56% (n = 14)	70% (n = 7)
Prophylaxis administered within 14 hours	80% (n = 41)	100% (n = 51)	56% (n = 14)	20% (n = 2)
Prophylaxis reviewed within 24 hours	78% (n = 40)	98% (n = 50)	24% (n = 6)	40% (n = 4)

At JCUH, VTE risk assessment within 14 hours was maintained at 100% across both cycles. Timely administration of prophylaxis within 14 hours improved from 80% (n = 41) to 100% (n = 51), and 24-hour prophylaxis review increased from 78% (n = 40) to 98% (n = 50).

At UHNT, VTE risk assessment improved from 56% (n = 14) in cycle one to 70% (n = 7) in cycle two. Prophylaxis review within 24 hours also increased from 24% (n = 6) to 40% (n = 4). In contrast, timely administration of prophylaxis within 14 hours declined from 56% (n = 14) to 20% (n = 2).

Documentation and patient communication

Documentation and communication outcomes are summarised in Table 2.

**Table 2:** Compliance with VTE Prophylaxis Guidelines Regarding Documenting and Patient Information by Hospital and Audit Cycle

Table 2	JCUH cycle one	JCUH cycle two	UHNT cycle one	UHNT cycle two
Documented discussion	12% (n = 6)	49% (n = 25)	8% (n = 2)	20% (n = 2)
Patient recall of discussion	14% (n = 7)	27% (n = 14)	24% (n = 6)	20% (n = 2)
Leaflet provided	0% (n = 0)	10% (n = 5)	0% (n = 0)	0% (n = 0)

At JCUH, documentation of prophylaxis discussions increased from 12% (n = 6) in cycle one to 49% (n = 25) in cycle two. Patient recall of discussions increased from 14% (n = 7) to 27% (n = 14), and patient information leaflets were provided to 10% (n = 5) of patients in cycle two.

At UHNT, documentation improved from 8% (n = 2) to 20% (n = 2). Patient recall of discussions remained limited across both cycles, and no patient information leaflets were distributed.

Patient preferences and dietary beliefs

Across both cycles, most patients reported that it was important for prescribed medications to align with their dietary or religious beliefs (cycle one: 96%, n = 72; cycle two: 98%, n = 60). Acceptance of non-compliant medication varied by clinical context. While 66% (n = 48) of patients in cycle one and 74% (n = 45) in cycle two reported willingness to accept non-compliant medication in life-threatening situations, acceptance declined for prophylactic indications, with only 46% (n = 33) in cycle one and 57% (n = 35) in cycle two indicating willingness.

Expectation of alternative options remained high, with 91% (n = 66) in cycle one and 100% (n = 61) in cycle two reporting that suitable alternatives should be offered where available. Among patients prescribed tinzaparin across both cycles (n = 72), 94% (n = 68) expressed a preference for dietary-compliant medication, and 92% (n = 66) reported that they would have preferred a non-porcine alternative.

Clinician awareness and practice

**Figure 3:**
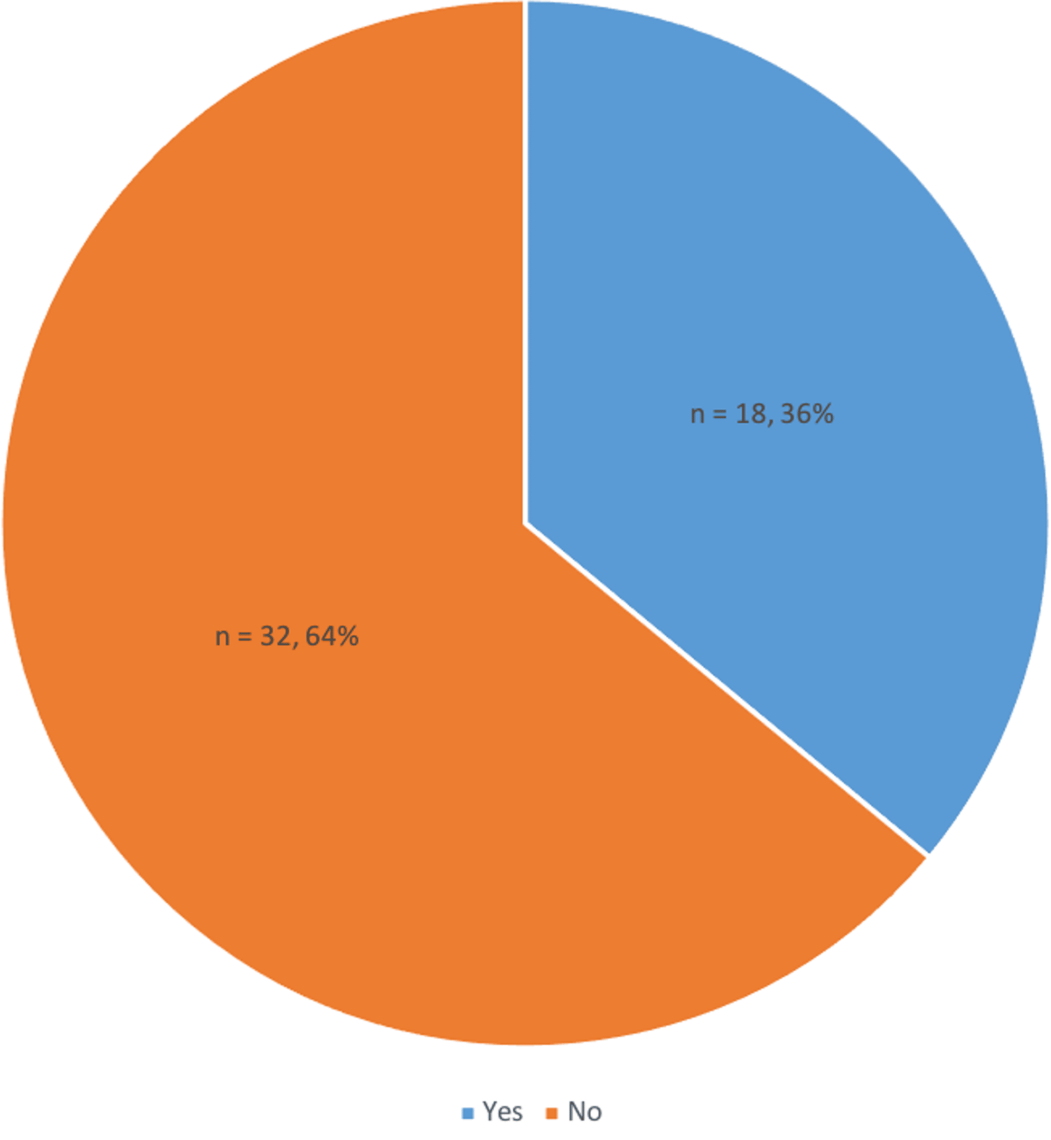
Proportion of Doctors Unaware That Tinzaparin Is Porcine-Derived

From our study, 64% (n = 32) of the doctors were not aware of the porcine origin of tinzaparin (Figure 3). Analysis showed that approximately 94% (n = 47) of doctors did not inform patients about the animal origin of tinzaparin, and 78% (n = 39) were not aware of alternative non-porcine options. A significant number of doctors (69%, n = 35) do not routinely inform patients on the risks and benefits of VTE prophylaxis. Additionally, there was a clear consensus (98%, n =49) among doctors that it is important for patients to be able to make informed choices about their medication. The most cited barriers to providing information were time constraints and lack of knowledge, highlighting a need for improved educational resources and support for healthcare professionals.

**Figure 4:**
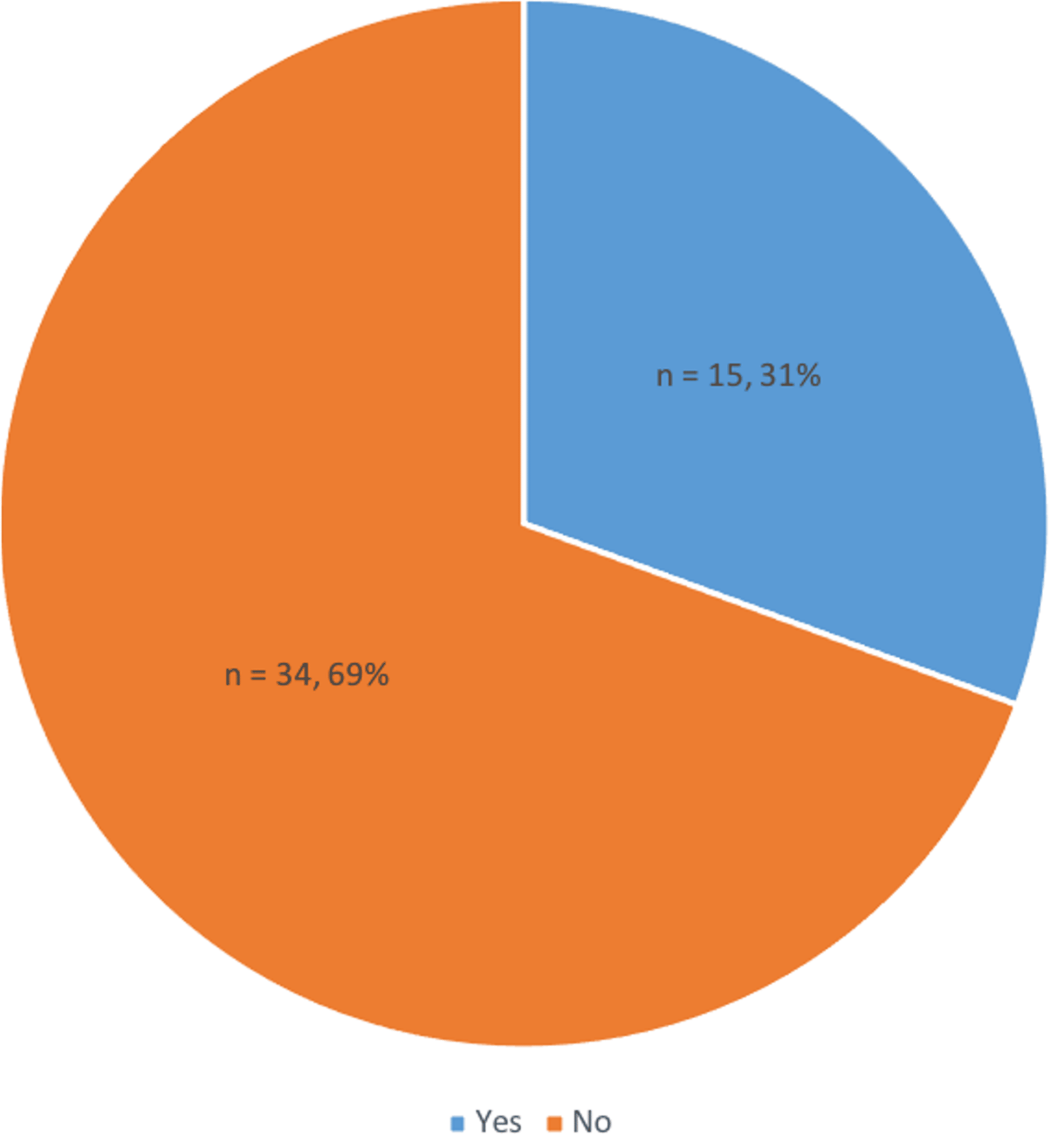
Percentage of Doctors Who Counsel Patients About VTE Prophylaxis VTE: Venous thromboembolism

The majority of doctors (64%, n = 32) do not inform patients on the risks and benefits of VTE prophylaxis before prescribing, as advised by NICE guidelines (Figure 4). Thirty-four percent (n = 17) of doctors attribute this to a lack of knowledge and 32% (n = 16) state this is due to time constraints. Other reasons such as no trust protocols outlining LMWH alternatives and not deeming this a high priority task were mentioned.

**Figure 5:**
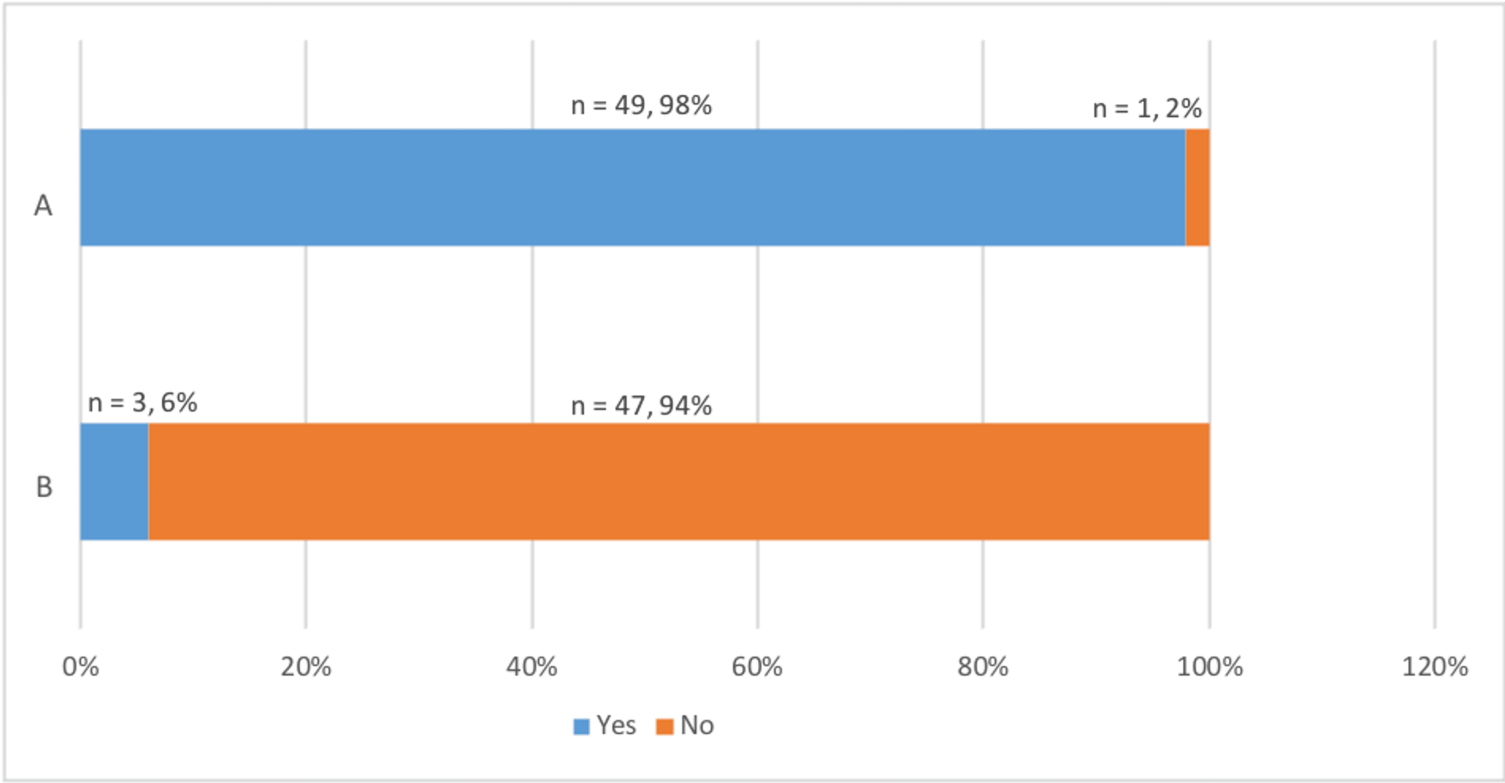
Proportion of Doctors Supporting Patient Autonomy and Counselling on LMWH Content A: Clinicians' views on the importance for patients to be able to make medical decisions based on their dietary preference/religious beliefs B: Clinicians asked if they counsel patients on the animal origin of Tinzaparin and other LMWHs LMWHs: Low-molecular-weight heparins

Figure 5A shows that a majority of doctors, around 94% (n = 47), do not inform patients about the animal origin of tinzaparin, indicating a lack of awareness about the medication’s porcine content. However, 98% (n = 49) of doctors recognised the importance of patients making informed choices about their medications (Figure 5B), demonstrating a strong consensus on the value of patient autonomy.

**Figure 6:**
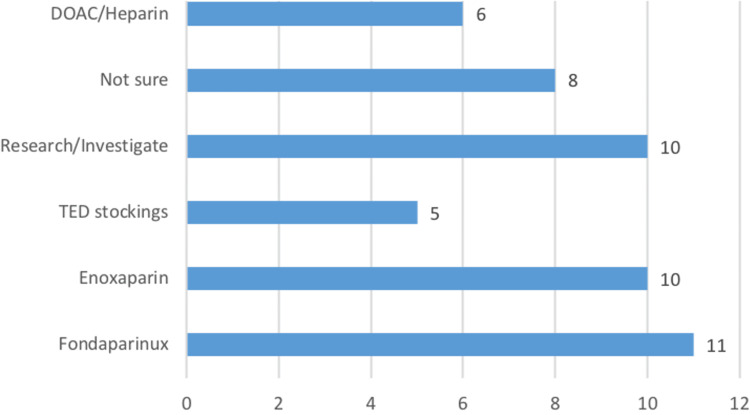
VTE Prophylaxis Choices by Doctors as an Alternative to Porcine-Derived LMWHs VTE: Venous thromboembolism; LMWHs: low-molecular-weight heparins; DOACs: direct oral anticoagulants

Furthermore, Figure 6 shows that 66% (n = 33) of doctors were unaware of a pharmacological non-porcine alternative and 20% (n = 10) offered another VTE prophylaxis that was porcine derived. This highlights a potential area for educational improvement.

## Discussion

In contrast, while UHNT demonstrated improvements in risk assessment and prophylaxis review rates, a decline in timely prophylaxis administration was observed. Review of local clinical practice indicated that electronic reminder systems had previously been withdrawn owing to concerns regarding disruption to emergency prescribing workflows, thereby limiting the effectiveness of digital alerts. Additional contextual challenges at UHNT included the absence of patient information leaflets, ongoing staffing pressures, and variable engagement at the ward level. These findings highlight the importance of tailoring quality improvement interventions to local infrastructure and workflow and suggest a need to re-evaluate mechanisms to support timely VTE prophylaxis, whether through reconsideration of digital reminders, as utilised at JCUH, or alternative strategies.

A significant finding of this audit relates to patient perspectives on medication compliance and dietary beliefs. Across both cycles, patients consistently emphasised the importance of culturally sensitive prescribing. Quantitative data from cycle one further contextualise this issue. Of the 76 patients reviewed, 47 (62%) were prescribed tinzaparin, a porcine-derived LMWH. Among these patients, 94% reported a preference for medications that complied with their documented dietary requirements. Of those expressing this preference, 92% stated that they would have preferred a non-porcine alternative; however, such an alternative had not been prescribed. This discrepancy highlights a substantial gap between patient preferences and prescribing practice, particularly in the context of prophylactic therapy where clinically acceptable non-porcine alternatives exist.

While many patients expressed willingness to accept non-compliant medications in life-threatening situations, there was marked reluctance to do so for prophylactic indications. This distinction underscores the necessity for early and transparent communication between clinicians and patients, particularly when prescribing medications derived from animal products. Patients with dietary restrictions consistently reported a desire for clarity regarding medication composition and a preference for non-porcine alternatives where available. These preferences align with the principles outlined in Good Medical Practice and NICE NG89 guidance, which emphasise respect for patient beliefs, shared decision-making, and informed consent [2,10]. Failure to provide appropriate counselling or access to acceptable alternatives may risk reduced adherence to VTE prophylaxis. Empowering patients through verbal counselling, written materials, or community engagement may foster trust and contribute to a more collaborative patient-clinician relationship [5].

Doctor awareness was assessed through a single questionnaire administered at the end of cycle one, which identified limited knowledge regarding the porcine origin of commonly prescribed low molecular weight heparins and a lack of formal training on available non-porcine alternatives. These findings directly informed the design of subsequent interventions, including targeted teaching sessions, informational posters, and prompts encouraging informed consent. Although a repeat clinician questionnaire was not conducted in cycle two, observed improvements in documentation and increased prescribing of non-porcine alternatives suggest a potential shift in awareness and prescribing behaviour following educational interventions. Nevertheless, in the absence of repeat questionnaire data, this inference should be interpreted with caution.

Limitations

This audit has a number of limitations. Clinician awareness and prescribing practices were assessed using a single questionnaire administered at the end of cycle one; the absence of a repeat clinician questionnaire in cycle two limits the ability to formally assess changes in knowledge or attitudes following the implemented interventions. In addition, the patient sample size at UHNT was relatively small in cycle two, which may limit the robustness of site-specific comparisons. Patient-reported outcomes relied on recall of discussions regarding VTE prophylaxis and medication composition, introducing the potential for recall bias [15]. Finally, this audit did not include a formal cost analysis of increased use of fondaparinux as a non-porcine alternative to LMWHs, which may be relevant for wider implementation and sustainability.

## Conclusions

This audit highlights that while targeted educational and system-level interventions can lead to measurable improvements in VTE prophylaxis compliance, continued progress in patient communication and culturally sensitive prescribing requires sustained efforts and reevaluation of current interventions. The outcomes at JCUH demonstrate that integrating electronic prompts, staff education, and structured documentation into routine practice can significantly enhance adherence to national standards and patient-centred care. In contrast, ongoing challenges at UHNT highlight a need for reflection on existing approaches and consideration of alternative or sustained interventions over longer timeframes.

To our knowledge, this is the first multi-site audit in the North East of England to evaluate culturally sensitive prescribing practices in the context of VTE prophylaxis. To build on this progress, future efforts should consider the implementation of mandatory dietary prompts within electronic prescribing systems, standardised scripts for informed VTE counselling, and widespread dissemination of multilingual patient information leaflets. Continued partnership with community and faith-based organisations will also be essential in empowering patients to engage in informed discussions about their treatment options. Ultimately, acknowledging and accommodating patients’ dietary beliefs is not merely a matter of cultural sensitivity; it is a critical component of safe, ethical, and inclusive healthcare delivery.

## Figures and Tables

**Figure 7:**
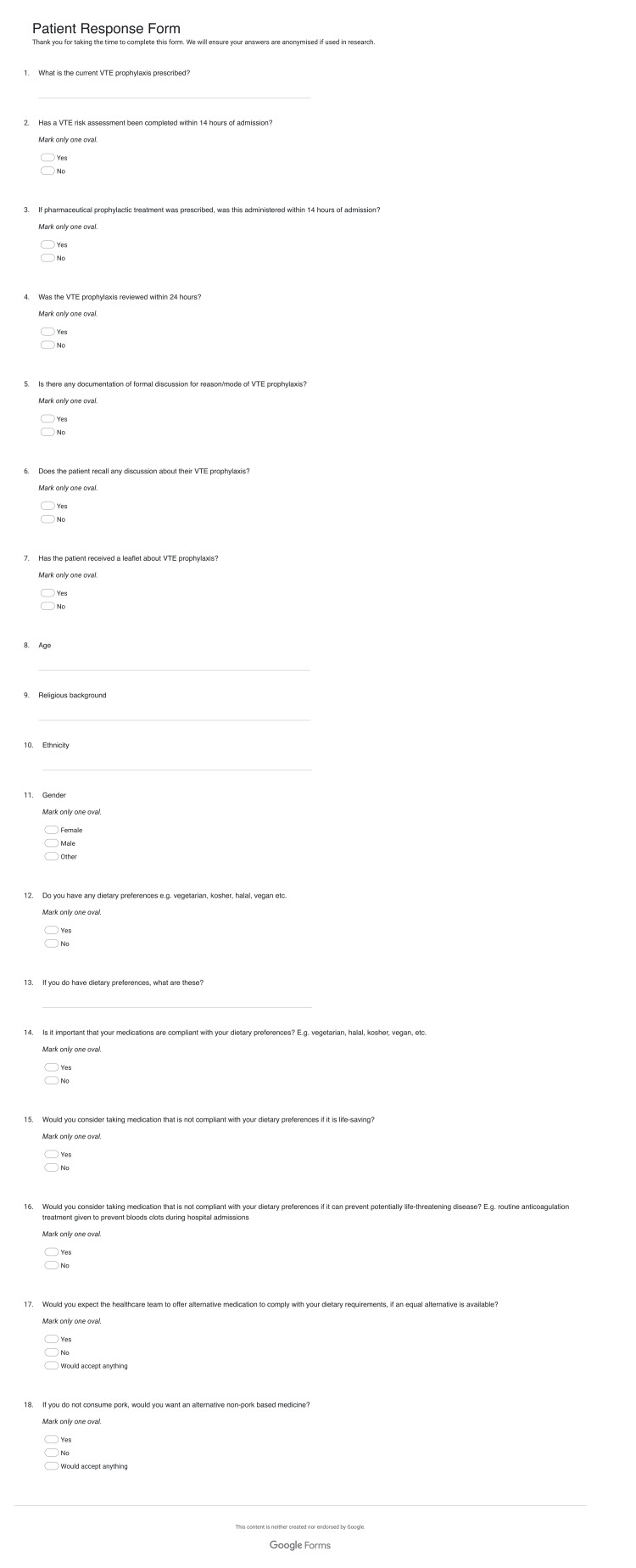
Patient Response Form The patient questionnaire was filled out with the auditor asking questions and inputting patient responses. In-person questioning allowed clarification of sections as some questions could be sensitive. All results were anonymised and analysed on Microsoft Excel.

**Figure 8:**
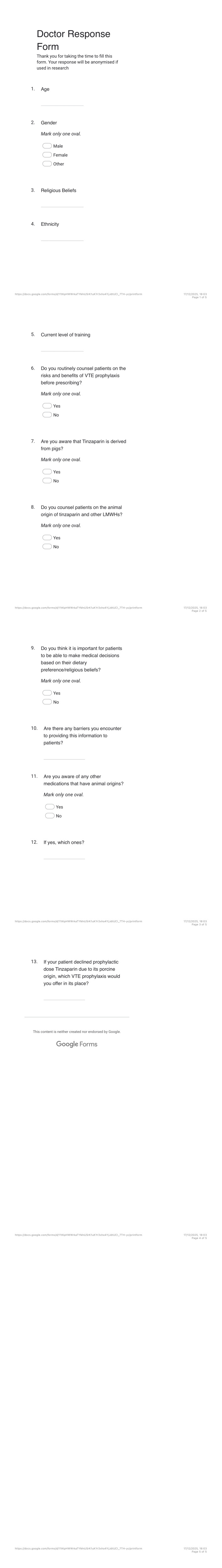
Doctor response form Google form questionnaire sent out online to all clinicians in both trusts via email and group chats. All results were anonymised and analysed on Microsoft Excel.
